# Macroscopic T-Wave Alternans: Unusual Presentation in a Cirrhotic Patient

**DOI:** 10.7759/cureus.79840

**Published:** 2025-02-28

**Authors:** Deepa Soodi, Vinod K. Reddy Cirra, Param Sharma

**Affiliations:** 1 Cardiology, Marshfield Clinic Health System, Marshfield, USA; 2 Internal Medicine, Deccan College of Medical Sciences, Hyderabad, IND

**Keywords:** cardio vascular disease, liver cirrhosis, long qt, t-wave alternans, ventricular arrhythmia

## Abstract

T-wave alternans (TWA), also known as repolarization alterations, refers to the alternating pattern observed in the ST segment, T wave, and U wave. Macroscopic TWA (MTWA) has been observed in patients with various conditions, such as ischemia, electrolyte imbalances, long QT syndrome, and transitions from tachycardia to a normal rhythm. Its recognition is of utmost importance as it serves as a red flag for potentially life-threatening cardiac arrhythmias. Prompt treatment is crucial in order to prevent cardiac arrest and its complications. Patients who exhibit MTWA should be promptly treated with intravenous magnesium sulfate, as magnesium deficiency can contribute to arrhythmias. Additionally, efforts should be made to identify and address any reversible causes that may be contributing to the MTWA. Timely intervention is key to achieving the best possible outcome for patients with MTWA.

## Introduction

T-wave alternans (TWA), also known as repolarization alterations, refers to the alternating pattern observed in the ST segment [1], T wave, and U wave [2]. Initially, obvious TWA was reported in the early 1900s during episodes of tachycardia, primarily attributed to ischemic causes [3]. Subsequently, visible TWA has been observed in patients with various conditions, such as ischemia, electrolyte imbalances, long QT syndrome, and transitions from tachycardia to a normal rhythm. In this case, we present an instance of macroscopic TWA (MTWA) associated with a prolonged QTc interval in the presence of electrolyte abnormalities in a cirrhotic patient.

## Case presentation

A 32-year-old male with a past medical history of anxiety and depression, along with chronic alcohol abuse, presented to the emergency room (ED) with complaints of weakness, low appetite, altered mental status, ongoing hematemesis, and melena. In addition, the patient presented with hypotension, with systolic blood pressure readings ranging from the 80s to 90s in the ED and demonstrating Kussmaul breathing pattern. The patient’s rectal temperature was measured at 34.3°C. 

Upon evaluation, the patient was found to have severely low blood sugar levels in the 30s, indicating hypoglycemia. The laboratory workup revealed several abnormalities, including a sodium level of 120 mmol/L, a potassium level of 2.5 mmol/L, a bicarbonate level of 6 mmol/L, and an anion gap of 44. Other findings included an elevated blood urea nitrogen (BUN) level of 44 mg/dL, creatinine level of 2.5 mg/dL, hemoglobin level of 7.8 g/dL, international normalized ratio (INR) of 2.7, elevated venous lactate level of 21.5 mmol/L, elevated ammonia level of 71 µmol/L, bilirubin level of 3.1 mg/dL, and mildly elevated aspartate aminotransferase (AST) level of 84 units/L. The patient received intravenous potassium and magnesium replacements, as well as intravenous thiamine and folate. Table 1 shows the patient's normal lab values.

**Table 1:** Patient’s lab values and normal reference values

Variables	Patient lab values	Normal lab values
Blood sugar	32 mg/dL	70-99 mg/dL
Sodium	120 mmol/L	133-144 mmol/L
Potassium	2.5 mmol/L	3.4-5.1 mmol/L
Bicarbonate	6 mmol/L	21-33 mmol/L
Anion gap	44 mmol/L	2-16 mmol/L
Blood urea nitrogen (BUN)	44 mg/dL	6-24 mg/dL
Serum creatinine	2.5 mg/dL	0.60-1.20 mg/dL
Hemoglobin	7.8 g/dL	11.7-15.5 g/dL
International normalized ratio (INR)	2.7	0.9-1.1
Venous lactate	21.5 mmol/L	0.5-2.0 mmol/L
Ammonia	71 µmol/L	16-53 µmol/L
Bilirubin	3.1 mg/dL	0-1.0 mg/dL
Aspartate aminotransferase	84 units/L	13-39 units/L
Magnesium	1.7 mg/dL	1.7-2.4 mg/dL
High sensitivity troponin	21 ng/L	0-57 ng/L

Hospital course and treatment received

The patient received intravenous fluids (IVF), two units of packed red blood cells (PRBC), intravenous pantoprazole, octreotide, and ceftriaxone, which were given due to concerns of gastrointestinal (GI) bleeding and variceal bleeding. Additionally, due to hypothermia, hypotension, and elevated venous lactate, a broad-spectrum antibiotic was initiated due to concerns of sepsis. Norepinephrine was also started, as the patient’s hypotension persisted despite resuscitation with IV fluids and PRBC. The patient underwent an esophagogastroduodenoscopy (EGD), which led to the diagnosis of an ischemic esophagus. At the same time, the patient developed acute kidney injury (AKI) and required hemodialysis as a treatment measure for this condition. The patient’s hypokalemia persisted throughout their hospitalization.

**Figure 1:**
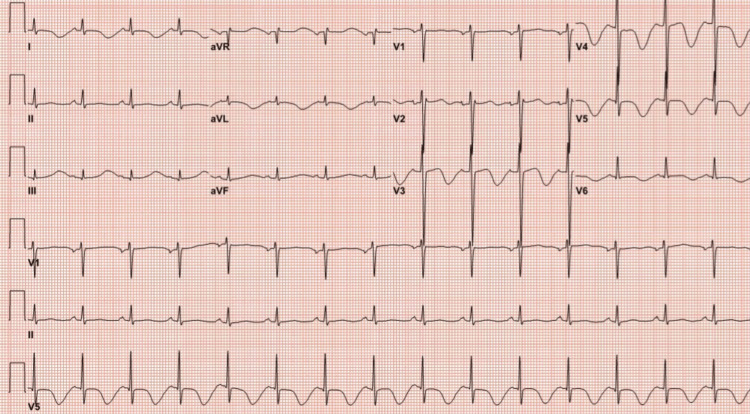
Electrocardiogram (EKG) EKG showing sinus rhythm with a heart rate of 90 beats per minute, T-wave inversions in the anterior leads, and the corrected QT interval (QTc) prolonged to 650 ms.

Unfortunately, on day 29, the hospital course became complicated by a cardiac arrest episode due to ventricular tachycardia. The patient received two rounds of cardiopulmonary resuscitation (CPR) and one dose of epinephrine, which resulted in the restoration of spontaneous circulation (ROSC). Blood work conducted during the code revealed a hemoglobin level of 9.5 g/dL, potassium level of 6.3 mmol/L, sodium level of 132 mmol/L, magnesium level of 1.7 mg/dL, and a high sensitivity troponin I (TNI) level of 21 ng/L. To address the high potassium levels, the patient was given a potassium-lowering cocktail containing sodium bicarbonate, regular insulin, and dextrose 50%. Additionally, intravenous magnesium replacement was administered. Once the patient’s condition stabilized, an electrocardiogram (EKG) was performed (Figure 1).

**Figure 2:**
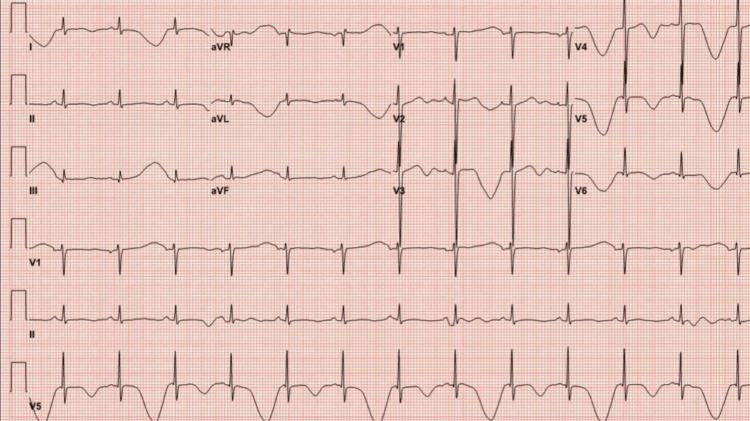
Electrocardiogram (EKG) EKG showing sinus rhythm with a heart rate of 78 beats per minute, prominent T-wave inversions in the anterolateral leads with alterations in amplitude, and the corrected QT (QTc) interval further prolonged to 802 ms.

A repeat EKG was conducted three hours later, as shown in Figure 2. These EKG findings indicated ongoing cardiac electrical abnormalities and prolonged ventricular repolarization.

**Figure 3:**
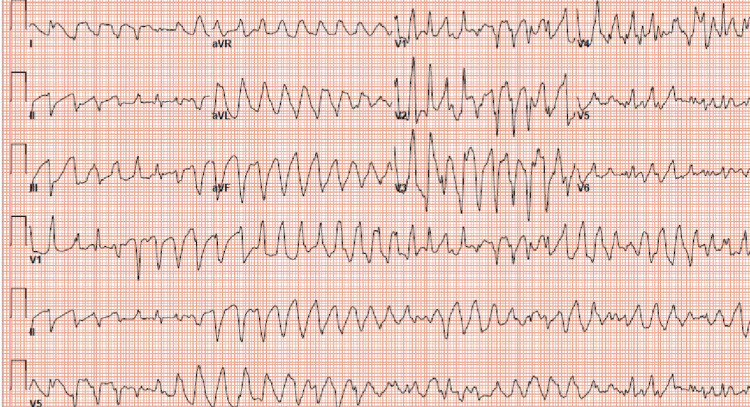
Electrocardiogram (EKG) EKG showing polymorphic ventricular tachycardia.

Despite efforts to correct the patient’s potassium and magnesium levels, he continued to experience episodes of polymorphic ventricular tachycardia (Figure 3), necessitating the administration of electrical shocks.

**Figure 4:**
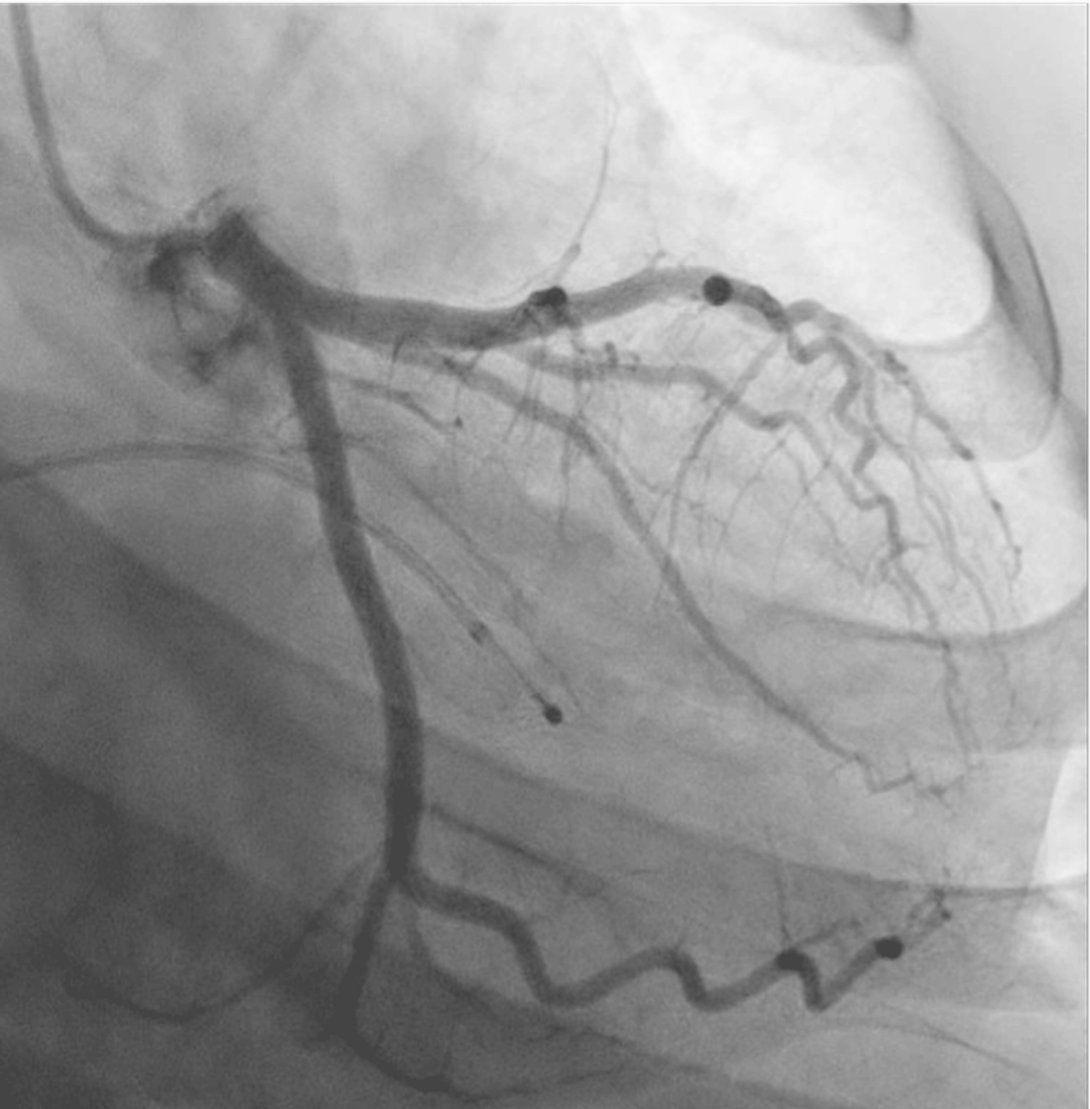
Left heart angiogram Left heart catheterization showing normal appearing left anterior descending (LAD) artery and left circumflex artery (LCx).

**Figure 5:**
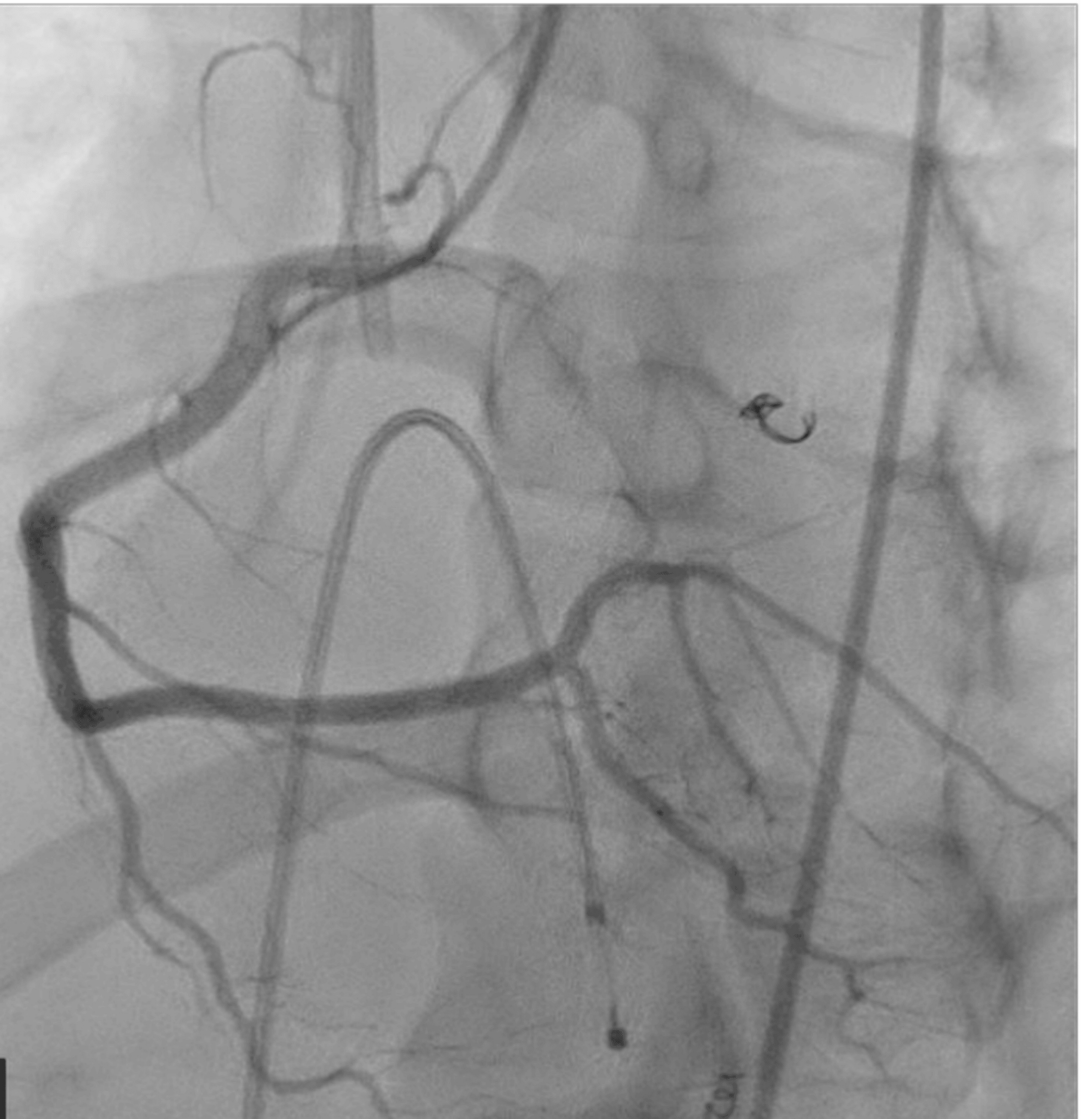
Left heart angiogram Left heart catheterization showing normal appearing right coronary artery (RCA).

To address this arrhythmia, the patient was initiated on isoproterenol at a rate of 2 mcg/min, which was then titrated to maintain a heart rate of 100 beats per minute, thereby shortening the QT interval and increasing the sinus rate. Subsequently, a temporary pacemaker was inserted to provide overdriving pacing. The patient also underwent a coronary angiogram, which revealed no significant stenosis (Figures 4, 5).

Measurement of the left ventricular end-diastolic pressure (LVEDP) indicated a value of 8 mmHg. A transthoracic echocardiogram was performed, showing a biplane left ventricular ejection fraction (LVEF) of 53% and no apparent regional wall motion abnormalities. However, abnormal septal motion consistent with a right ventricular pacemaker was observed. Throughout this period, the patient remained under sedation.

**Figure 6:**
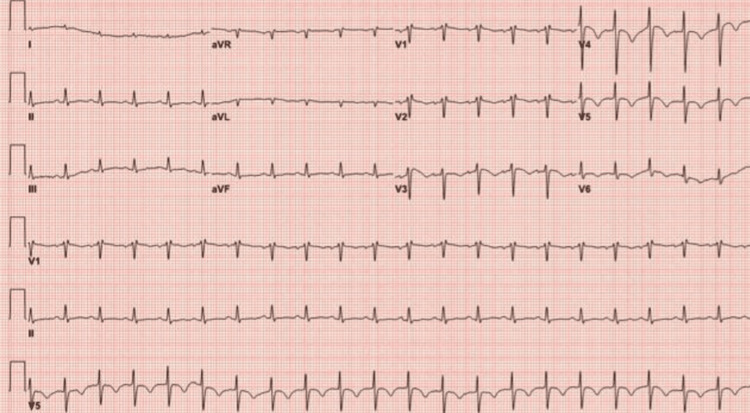
Electrocardiogram (EKG) EKG showing sinus tachycardia with a heart rate of 120 bpm and a corrected QT (QTc) interval of 562 ms, corrected for sinus tachycardia.

Medications were thoroughly reviewed to identify and avoid any potential drug interactions that could contribute to QT prolongation, which can increase the risk of arrhythmias. The medications loperamide and pantoprazole were discontinued, and efforts were made to maintain potassium levels above 4.5 mmol/L and magnesium levels above 2.4 mg/dL with frequent lab testing. The patient’s progress was closely monitored through serial EKGs. As shown in Figure 6, the QTc interval started to show improvement following the discontinuation of the medications and the maintenance of appropriate electrolyte levels. Subsequently, the temporary ventricular pacemaker (TVP) was removed.

The patient was then evaluated by an electrophysiologist to explore the possibility of congenital long QT syndrome. However, a review of the patient’s EKG from a few years prior to this admission did not demonstrate consistent findings with congenital long QT syndrome. Additionally, there was no family history of sudden cardiac death, structural heart disease, or unexplained syncopal episodes among the patient’s relatives.

Given the clinical presentation and findings, the most probable diagnosis for this patient is acquired long QT syndrome in the context of liver cirrhosis or end-stage liver disease. During the course of treatment, the patient’s renal function gradually improved, leading to the discontinuation of hemodialysis. However, due to the patient’s ongoing alcohol abuse, they were deemed ineligible for liver transplantation. Genetic testing was not performed as the patient was discharged home under hospice care and unfortunately passed away.

## Discussion

MTWA, or TWA, refers to the beat-to-beat variation in the amplitude and polarity of the T wave observed on a surface EKG. It occurs as a result of rapid changes in ventricular depolarization, as was recorded in this patient (Figure 2). There are multiple factors associated with the development of MTWA, including prolonged QT interval, electrolyte imbalances, and myocardial ischemia. The literature contains several case reports [4] documenting the presence of MTWA in patients with conditions, such as myocarditis associated with rheumatic heart disease and Wernicke’s encephalopathy. 

Our patient had a history of chronic alcoholic liver disease, end-stage liver failure, and cirrhosis. In cirrhotic patients, the precise mechanism of QT prolongation is not well understood. In one study, multivariate analysis showed that the Child-Pugh score and plasma norepinephrine concentration were independent factors that predicted QT prolongation [5]. Plasma norepinephrine is a marker of the sympathetic nervous system (SNS). Increased activity of the SNS is common in advanced cirrhosis, and its impact on cardiac function may be magnified by vagal denervation. Another possible mechanism of QT prolongation in cirrhotic patients is ion channel dysfunction [5]. In ventricular myocytes from rats with chronic bile duct ligation, the action potential was found to be prolonged, and the function of K+ channels responsible for ITO and IK currents was impaired [5]. Combined effects of potassium current defects and chronic adrenergic stimulation lead to action potential (and QT interval) prolongation. Impaired drug metabolism [6] is another explanation for QT prolongation. Studies have shown that approximately 60% of patients with advanced cirrhotic exhibit QT prolongation, regardless of its cause, and that this prolongation closely correlates with cirrhosis severity [7]. Hence, early diagnosis of alcoholic liver disease is important to encourage alcohol abstinence and minimize the progression of liver fibrosis and cirrhosis. Liver transplantation is the ultimate therapy for alcoholic cirrhosis, which mandates six months of alcohol abstinence [8]. Our patient chose hospice care and passed away shortly. 

Our patient had no prior history of cardiovascular disease or a family history of sudden cardiac death. However, he did have a history of end-stage liver disease, a condition known to be associated with autonomic dysfunction, poor drug metabolism, and prolonged QT intervals. Additionally, the patient was on multiple medications (fexofenadine, melatonin, nadolol, pantoprazole, IV vancomycin, ondansetron, and TPN for nutrition) due to prolonged hospitalization and had multiple comorbid conditions, which could have contributed to the prolongation of the QT interval. Upon reviewing the medications during the cardiac arrest episode, it was discovered that loperamide and pantoprazole, when taken in excess doses or in the presence of hypokalemia, increased the risk of torsades de pointes associated with QT prolongation [9]. Therefore, medications that have the potential to prolong the QT interval should be discontinued to minimize the risk. 

MTWA has been identified as an increased risk factor for sudden cardiac death in patients with heart failure and reduced ejection fraction, making it a significant negative predictor in this particular patient population [10]. In cases where a patient develops torsades de pointes due to QT prolongation, it is advisable to perform a coronary angiogram to exclude any underlying ischemic causes. It is also important to obtain a detailed family history to assess the possibility of congenital channelopathy and congenital long QT syndrome. However, in our patient’s case, he denied having any family history of sudden cardiac death, structural heart disease, or unexplained syncopal episodes. Additionally, his EKG from a few years ago did not indicate the presence of congenital long QT syndrome. Nevertheless, if there are any concerns regarding a potential congenital etiology, offering genetic testing would be warranted. 

Upon recognizing the pattern of prolonged QT interval, prompt treatment should be initiated. This includes administering intravenous magnesium sulfate to correct magnesium deficiency and addressing other electrolyte abnormalities. It is crucial to identify any medications that may contribute to QT interval prolongation and discontinue them. In addition, overdrive pacing with the infusion of isoproterenol can be considered as an emergent measure. Atrial or ventricular pacing can help prevent bradycardia and ventricular pauses. By increasing the heart rate, repolarization across the tissues can be better accommodated [11]. These interventions aim to restore normal cardiac electrical activity and prevent further complications.

## Conclusions

MTWA is a relatively uncommon finding in clinical practice. However, its recognition is of utmost importance as it serves as a red flag for potentially life-threatening cardiac arrhythmias. Prompt treatment is crucial in order to prevent cardiac arrest and its complications. Patients who exhibit MTWA should be promptly treated with intravenous magnesium sulfate, as magnesium deficiency can contribute to arrhythmias. Additionally, efforts should be made to identify and address any reversible causes that may be contributing to the MTWA. Addressing these factors and providing appropriate treatment can reduce the risk of cardiac arrest and other serious arrhythmias. Timely intervention is key to achieving the best possible outcome for patients with MTWA.
